# Temporomandibular joint disc plication with MITEK mini anchors: surgical outcome of 65 consecutive joint cases using a minimally invasive approach

**DOI:** 10.1186/s40902-020-00259-2

**Published:** 2020-04-29

**Authors:** Bu-Kyu Lee, Jun Hee Hong

**Affiliations:** 1 Department of Oral and Maxillofacial Surgery, Asan Medical Center, College of Medicine, University of Ulsan, 05505, Olympic-ro 88, 43-gil, Songpa-gu, Seoul, Republic of Korea; 2 Biomedical Engineering Research Center, Asan Institute for Life Sciences, Asan Medical Center, College of Medicine, University of Ulsan, 05505, Olympic-ro 88, 43-gil, Songpa-gu, Seoul, Republic of Korea

**Keywords:** Temporomandibular joint disorders, Disc displacement, Disc plication, MITEK anchor, TMJ surgery

## Abstract

**Background:**

The purpose of this study is to introduce our modified disc plication technique using MITEK mini anchors and to evaluate the clinical outcome for patients with internal derangement (ID) of the temporomandibular joint (TMJ).

**Patients and methods:**

We evaluated 65 joints in 46 patients, comprised 32 women and 14 men, who first visited the Asan Medical Center from December 2012 to December 2016. The age of the patients ranged from 14 to 79 years, with a mean age of 36.6 years. The patients presented with joint problems including pain, joint noise, and mouth opening limitation (MOL). Patients who met our inclusion criteria underwent unilateral or bilateral disc repositioning surgery with our minimally invasive disc plication technique using MITEK mini anchors and No. 2-0 Ethibond® braided polyester sutures. The variables taken into account in this study were the range of maximum mouth opening (MMO), painful symptoms (evaluated with the visual analog scale, VAS), and the type of noise (click, popping, crepitus) in the TMJ.

**Results:**

Preoperative examination revealed painful symptoms in 50.7% (*n* = 35) of the operated joints (*n* = 69) and the presence of clicks in 56.5% (*n* = 39). Postoperative examination revealed that 4.3% (*n* = 3) of the operated joints had painful symptoms with lower intensity than that in the preoperative condition. Additionally, 17.4% (*n* = 12) had residual noise in the TMJ, among which two were clicking and the other 10 had mild crepitus. The intensity of the postoperative residual noise was significantly decreased in all cases compared to that in the preoperative condition. Among patients with MOL below 38 mm (*n* = 18), the mean MMO was 31.4 mm preoperatively and 44.2 mm at 6 months postoperatively, with a mean increase of 13.8 mm. A barely visible scar at the operation site was noted during the postoperative observation period, with no significant complications such as facial palsy or permanent occlusal disharmony.

**Conclusion:**

Subjective symptoms in all patients improved following the surgery. TMJ disc plication using MITEK mini anchors with our minimally invasive approach may be a feasible and effective surgical option for treating TMJ ID patients who are not responsive to conservative treatment.

## Background

Internal derangement of the temporomandibular joint (TMJ ID) is the most common condition that causes temporomandibular joint disorders (TMDs) [1]. Reducible or non-reducible disc displacement of TMJ can result in noise or crepitus, arthritis, condyle head resorption, jaw deformities, open bite, inflammation, and joint pain [2]. Although some patients may lack visible symptoms of TMJ ID, the condition can affect normal jaw functions such as chewing, swallowing, and phonetics in most cases [1].

In 1979, McCarty and Farrar introduced a surgical technique for disc repositioning as a treatment option for TMJ ID, reporting a 94% success rate over a 6-year period [3]. Other studies have presented variations in the technique, with improved symptoms and different follow-up periods [1, 4, 5]. However, despite these reports, many surgeons have indicated that the reposed disc does not last long in its new position and the high success rate reported in the original study could not be achieved [5].

The reported clinical outcomes for TMJ disc repositioning surgery vary and are often unpredictable [5, 6]. Traditional disc repositioning techniques such as suturing inflamed and often degenerated ligaments result in disc instability and the inevitably wide skin incision results in a significant amount of scarring on the face [1]. Therefore, new surgical techniques have been developed for TMJ disc repositioning [1, 7–9].

Skeletal anchors are used in various surgical procedures to attach soft tissue to soft or hard tissues. They can also be used in orthopedic, reconstructive, and orbital procedures [10, 11]. In the field of TMJ surgery, Wolford et al. and other surgeons reported improved results by using this anchor system. According to these authors, the MITEK mini anchor (DePuy Mitek, Raynham, MA) showed long-term stability and successful results in disc repositioning surgery [1, 12]. This anchor system provides accurate and tight fixation of the disc to the head of the condyle, harmonizing the disc-condylar relationship when in use and improving the resistance of the disc to displacement caused by a pullout force from the masticatory muscles [13]. Thus, the concept of using a bone anchor and artificial ligaments for disc stabilization is attractive as it does not depend on the structural integrity of soft tissues to maintain postsurgical disc stability [1].

However, several oral and maxillofacial surgeons have reported that the effectiveness of TMJ disc repositioning is low [14]. In this context, some surgeons preferred non-invasive techniques like arthroscopy rather than invasive TMJ repositioning surgery to resolve TMJ ID [15]. However, for patients with refractory limitation of mouth opening or annoying popping of the disc accompanied by intermittent habitual luxation, adequate and comfortable mouth opening can only be achieved through appropriate surgical methods such as disc repositioning surgery via an open TMJ approach [16].

Recently, some surgeons reported that a specific technique using MITEK mini anchors often caused severe adhesion of the superior joint space, resulting in severe limitation of mouth opening compared to the conventional disc plication technique. The surgeons attributed this outcome to the posterior part of the disc remaining open after the junction with the retrodiscal tissue of the TMJ was excised. As a result of this preparatory procedure, severe scarring can develop in the dead space [14]. To overcome this technical hurdle, we modified the conventional technique by utilizing MITEK mini anchors.

The aim of this study is to introduce and evaluate our modified disc repositioning surgery technique using MITEK mini anchors, which improves the clinical outcomes. We also report the clinical data for patients who underwent disc repositioning surgery using our modified MITEK mini anchor technique.

## Patients and methods

This was a retrospective study evaluating the treatment records of 65 joints in 46 consecutive patients (32 women and 14 men) who underwent TMJ disc repositioning surgery during the period of December 2012 to December 2016. This clinical study was approved by the Institutional Review Board of Asan Medical Center (IRB number: S2018-1771-0001). The age of the patients ranged from 14 to 79 years, with a mean age of 36.6 years. The inclusion criteria for the study were as follows: [17] (1) American Society of Anesthesiology status I patients [18] (i.e., healthy patients) with TMJ disorders identified based on clinical examination [19] and magnetic resonance imaging (MRI); (2) no previous surgery involving the TMJ; (3) the presence of pain, joint noise, or limited mouth opening [20]; (4) treatment with the MITEK mini anchor; and (5) post-operative follow-up period of more than 6 months.

In our protocol, the indications for TMJ disc repositioning surgery were as follows: (1) painful anterior disc displacement with reduction that did not respond to nonsurgical and minimally invasive procedures, (2) anterior disc displacement without reduction with persistent pain and limited mouth opening that did not respond to nonsurgical and minimally invasive procedures, (3) severe TMJ sounds (ex. popping) with intermittent locking of the disc, (4) patient request for surgery after conservative treatment, (4) no serious systemic disease such as diabetes mellitus or rheumatoid arthritis, (5) no serious mental health condition, and (6) no previous TMJ surgery.

### MITEK mini anchor

**Fig. 1:**
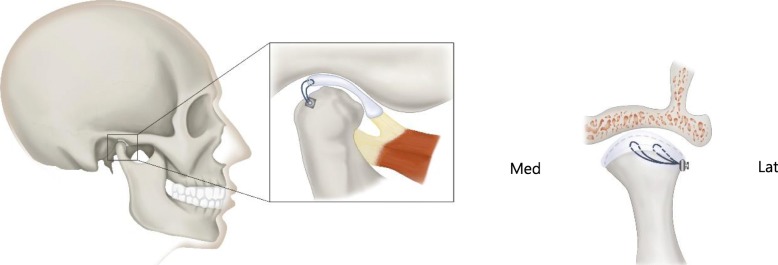
Schema of position of MITEK mini anchor (**a**) and placement of two No. 2-0 polyester sutures at posterior segment of TMJ disc sutured to mini anchor placed on most lateral superior and posterior aspect of mandibular condyle (**b**). Med, medial aspect of mandibular condyle; Lat, lateral aspect of condyle

MITEK anchors were first developed for orthopedic surgery such as shoulder cuff repair, medial and lateral collateral ligament repair, bicep tendon reattachment, and other muscle, ligament, and tendon repair surgery [11, 21]. The MITEK mini anchor is a suitable size for TMJ disc stabilization, and successful utilization of the anchor for TMJ disc repositioning has been reported by Wolford et al [1, 22]. The anchor consists of a titanium alloy shaft with a 2-0 Ethibond® braided polyester suture threaded through its eyelet and wings. The shaft is made of 90% titanium metal alloy, 6% aluminum, and 4% vanadium. The two retention wings are made of nickel and titanium. The composition and structure of the MITEK mini anchor are known to contribute significantly to the osseointegration of the anchors in the condyle, proper positioning of the TMJ disc, and long-term stability of the surgery [13, 23]. The general scheme of disc plication using MITEK mini anchor was illustrated in Fig. 1.

### Surgical procedures

**Fig. 2:**
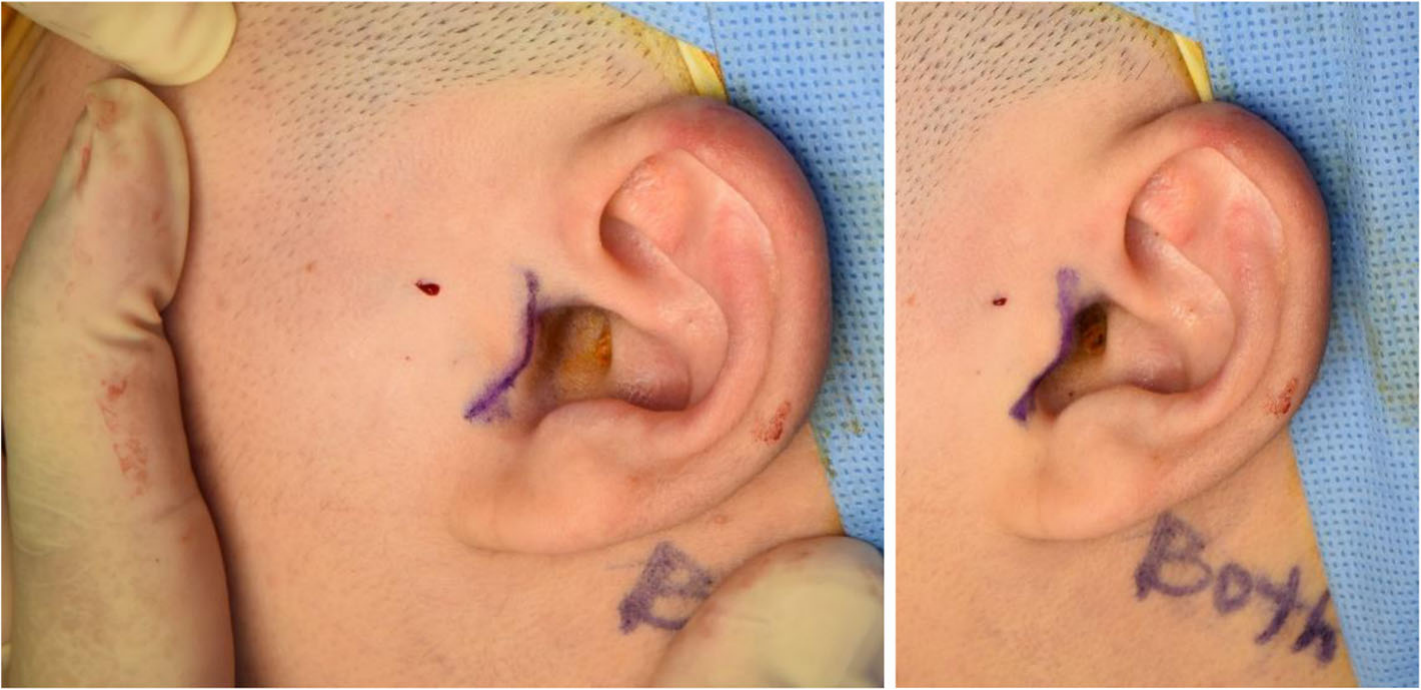
Anterior digital traction for initial surgical markings with methylene blue. **a** Modified short endaural approach marked with methylene blue when digital traction is released **b** showing how the incision is hidden

**Fig. 3:**
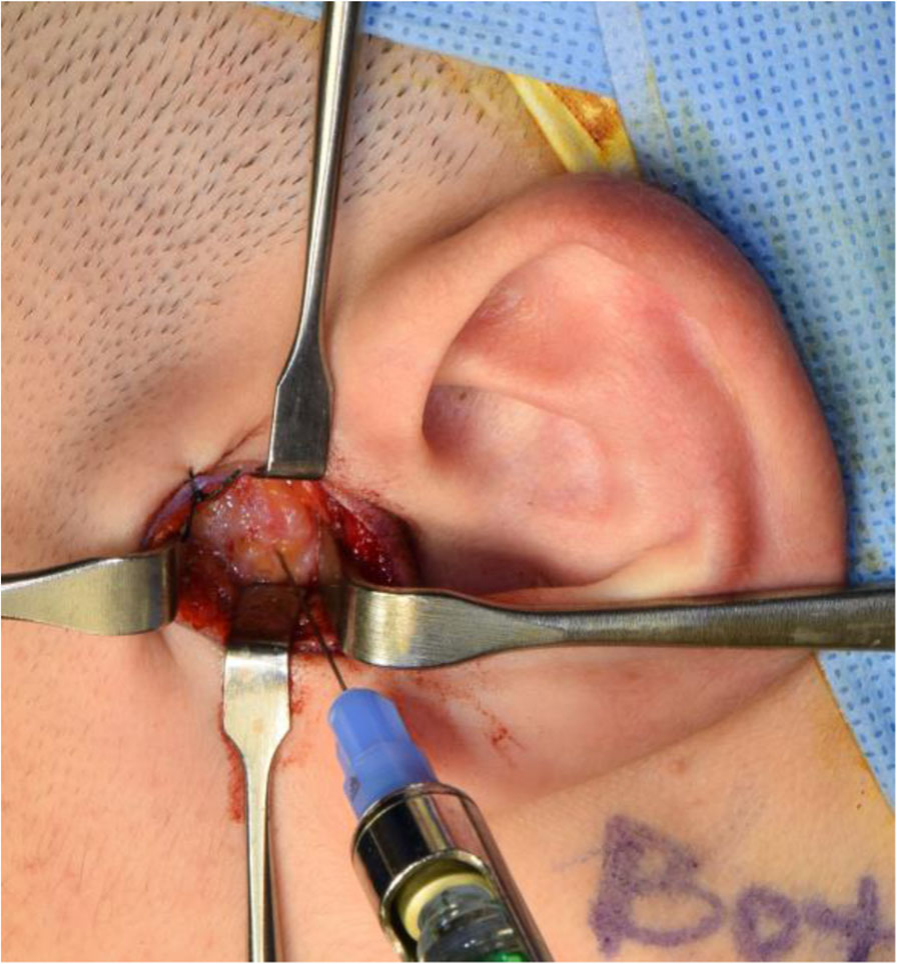
Intra-articular injection of lidocaine (2% with 1:100,000 epinephrine) to facilitate further dissection of capsular ligament

**Fig. 4:**
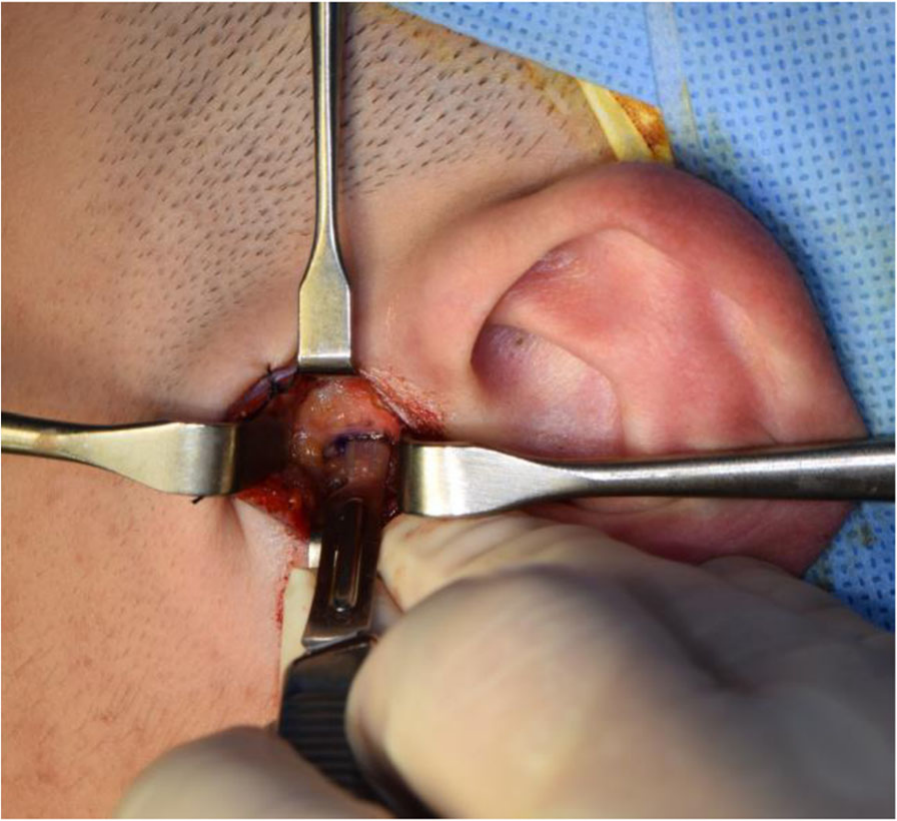
Horizontal incision of capsular ligament after the confirmation of condylar position. This layer is tagged with two 5-0 white Vicryls for ease of layer-by-layer suture afterwards

**Fig. 5:**
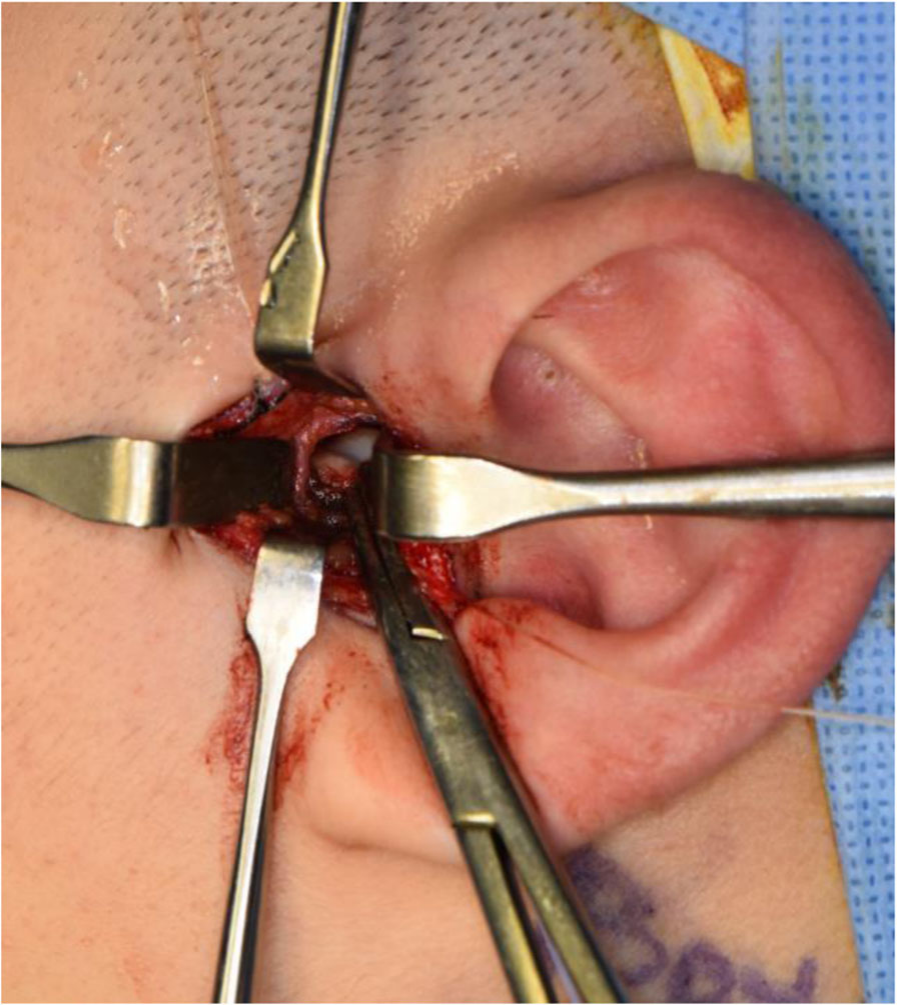
Displaced disc is detached from fibrous adhesion and rotated posterolaterally to achieve the correct condyle-disc-fossa relation

**Fig. 6:**
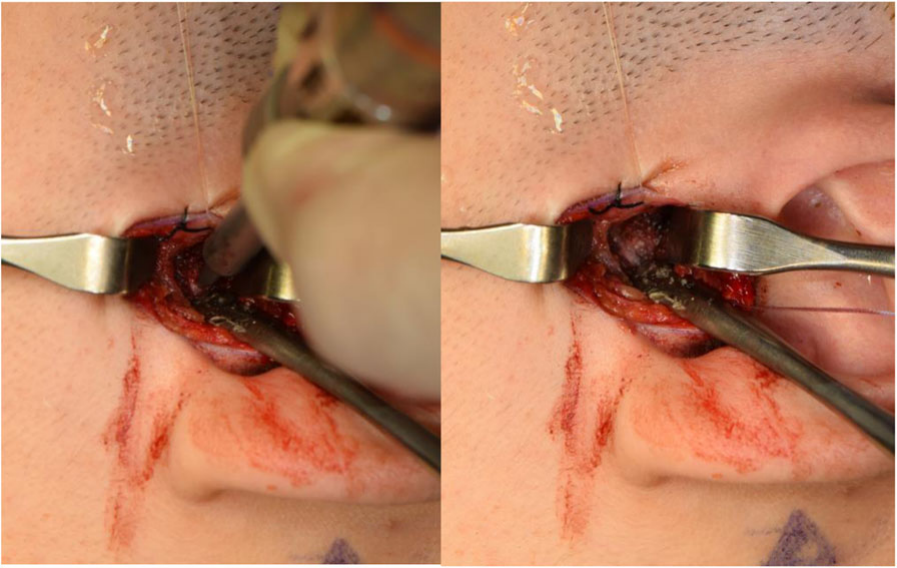
**a**, **b** Initial bone perforation for MITEK mini anchor implant placement located at most lateral, superior, and posterior aspect of mandibular condyle

**Fig. 7:**
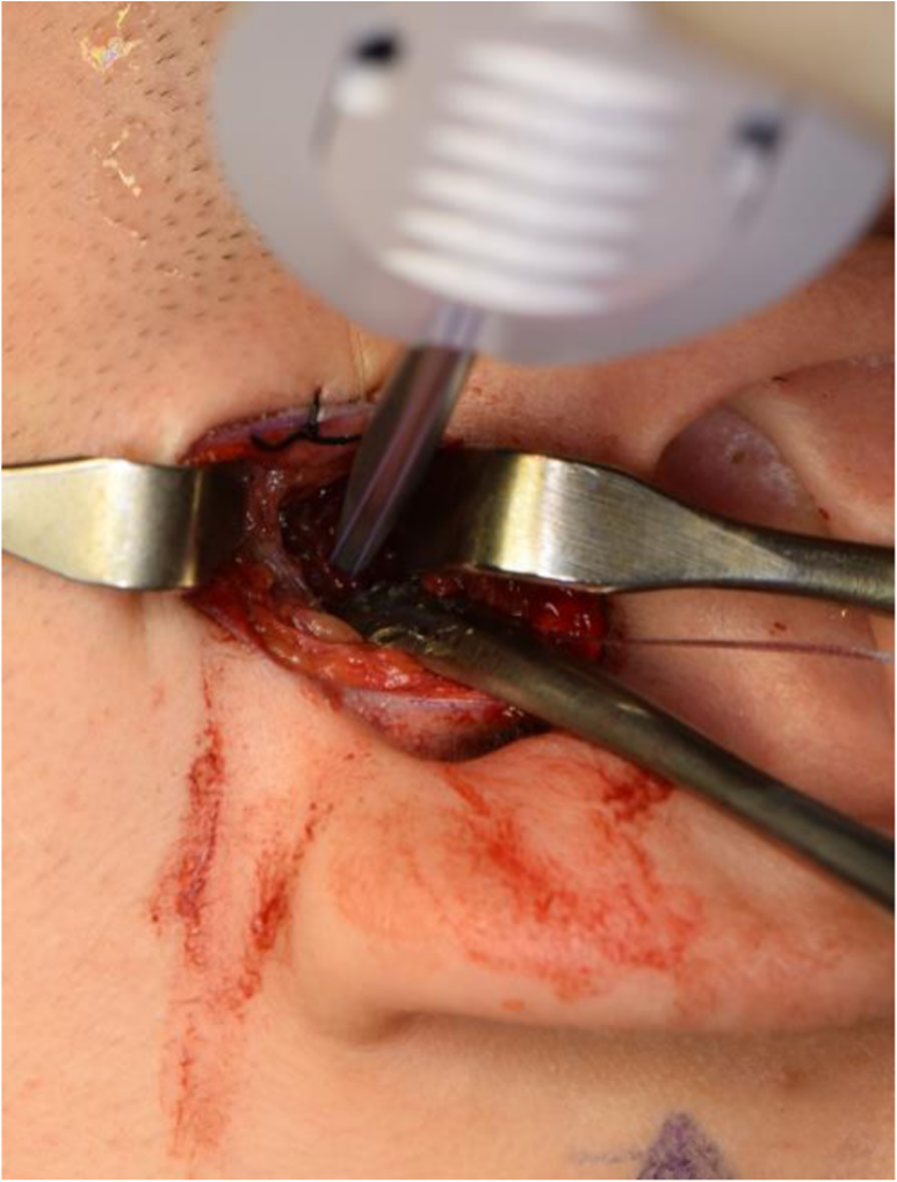
MITEK mini anchor placement at most lateral, superior, and posterior aspect of mandibular condyle

**Fig. 8:**
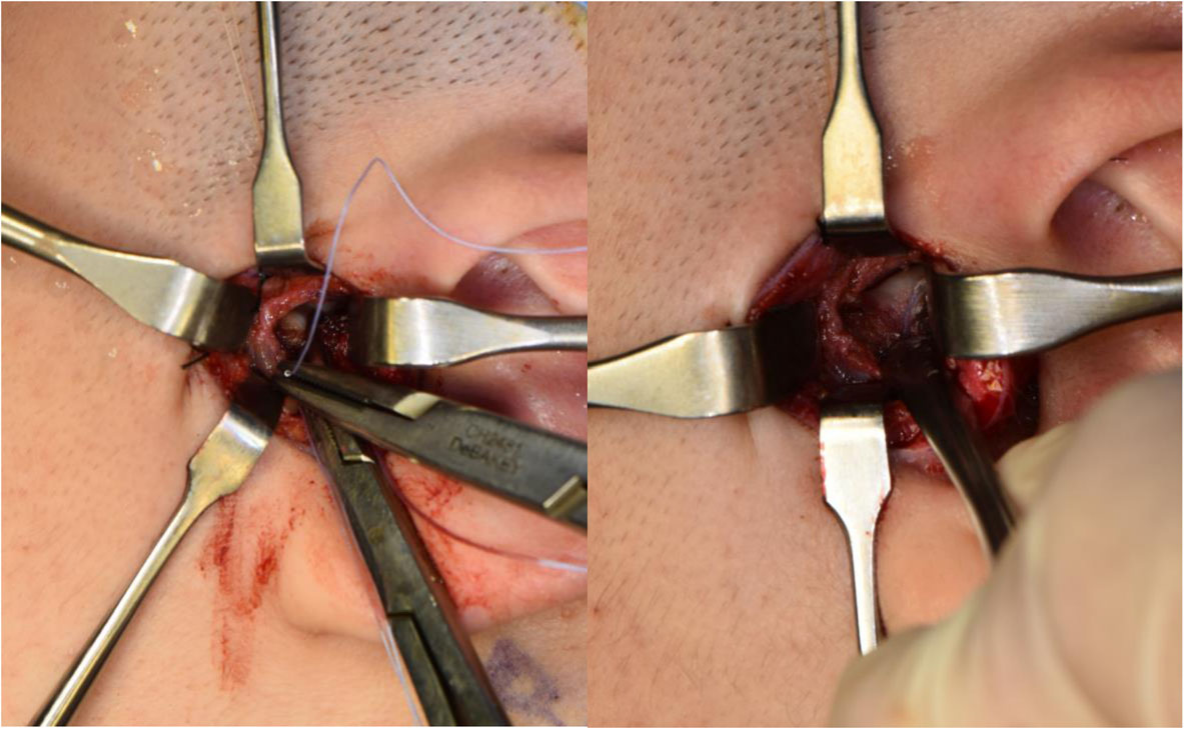
**a**, **b** Location of TMJ disc plication with No. 2-0 polyester suture to MITEK mini anchor

**Fig. 9:**
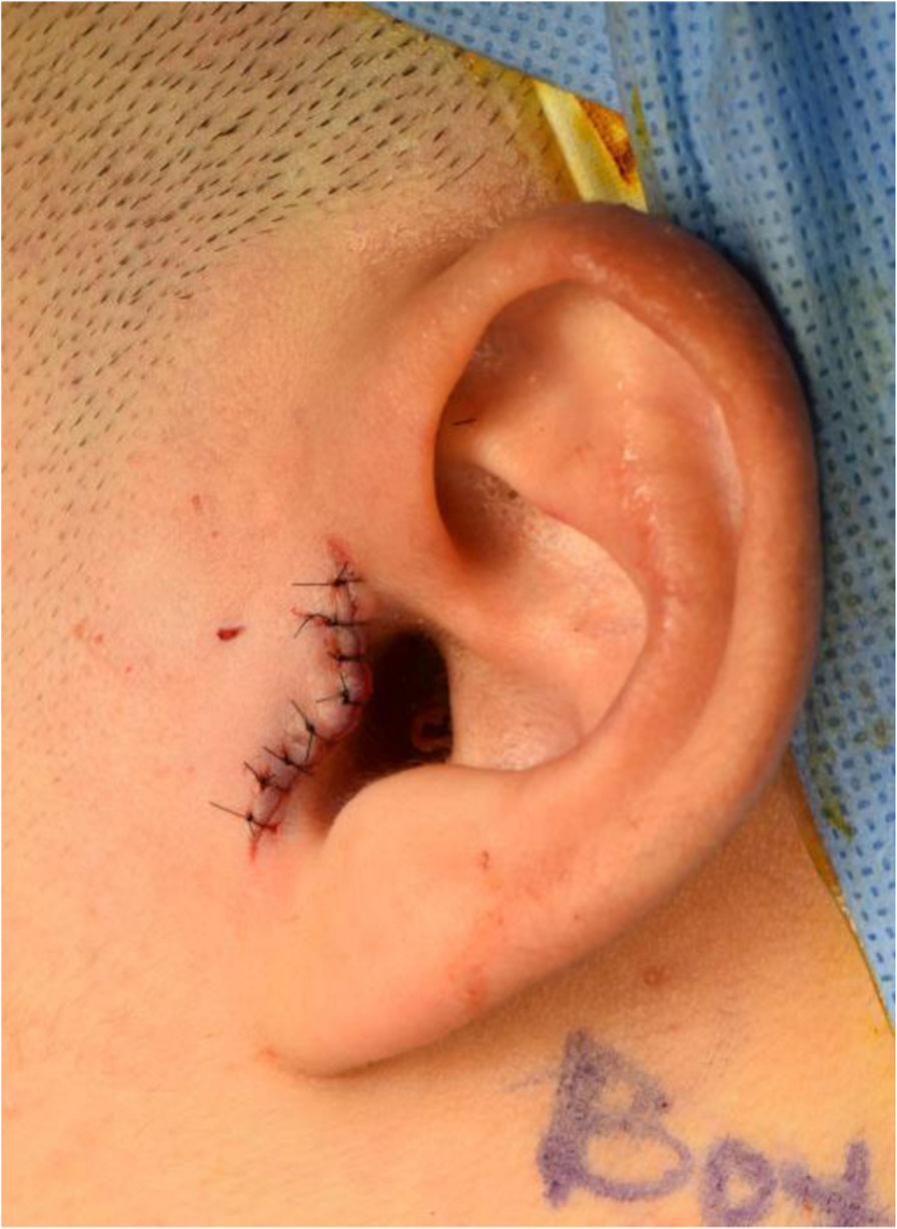
Final flap repositioning and placement of 8 No. 6-0 nylon single interrupted sutures

A meticulous surgical intervention is needed to obtain predictable results. All patients received the surgery with the same procedures (Figs. 2, 3, 4, 5, 6, 7, 8, and 9). All patients underwent surgery under general anesthesia with nasotracheal intubation, and sterile surgical preparation and draping were performed in a routine manner for all patients. With digital traction of the preauricular region, a short endaural incision line was drawn with a sterilized marking pen. Unilateral or bilateral preauricular sites were injected with 2.0 ml of lidocaine (2% with 1:100,000 epinephrine) in a subcutaneous plane. Using a No. 15 scalpel, incisions were made along the surgical design line. Dissection was performed with sharp dissection scissors and a Bovie electrocautery from the tragal cartilage down and forward approximately 15 to 20 mm through the subcutaneous tissue. The first assistant was frequently instructed to pull the mandible anteriorly and to push backward posteriorly. Afterward, the surgeon placed his point finger on the surgical area and determined the position of the mandibular condylar head and the articular eminence based on the tactile sensation. This step of the procedure enabled the surgeon to approach the joint space safely even with a small incision. Blunt dissection was performed with mosquito forceps in the direction of the superficial temporal fascia and below the fat tissue to avoid damage to the facial nerves. The superficial temporal fascia was dissected with mosquito forceps and separated using Bovie electrocautery. Using Senn-Miller retractors, the first assistant entered the dissected plane and retracted the forceps in an anterior and inferior direction. The second plane was elevated and dissected in the same fashion, arriving at the temporal fascia at this level to reveal the TMJ capsule. One milliliter of lidocaine (2% with 1:100,000 epinephrine) was injected into the superior joint space to hydraulically displace the disc inferiorly. The lateral capsule was incised horizontally with a No. 15 scalpel. Dissection of the superior joint space was performed with a periosteal elevator, enabling entrance into the superior joint space. Disc liberation enabled posterior movement and repositioning of the TMJ disc. It was often necessary to free the disc anteriorly when the ligament adhered to the anterior band of the disc to the anterior slope of the articular eminence. The medial attachments sometimes had to be released as well. Using a MITEK drill bit (2.1 mm in diameter), a hole was made laterally in the posterior head of the condyle. The mini anchor was inserted on the most posterior aspect of the mandibular condyle. The position of the anchor varied slightly for each case, but was generally 5 to 10 mm below the superior aspect of the condyle. TMJ disc plication was achieved using two No. 2-0 Ethibond® braided polyester sutures placed on the posterior part of the disc. After the discs were repositioned with sutures, the condyle was manipulated in various directions to confirm the disc and condylar unit could move harmoniously and the disc was well-secured in its new, optimal position. We usually coagulated the retrodiscal tissue and the posterior bilaminar area. To better secure the reposed disc, additional sutures were added to the posterolateral margin of the disc and internal capsule of the TMJ, as with the conventional method. Afterward, the surgical site was irrigated with normal saline and the lateral capsule was sutured back together. No. 5-0 Vicryl sutures (Ethicon, Somerville, NJ) were used to reposition the surgical planes. Adequate and accurate repositioning and suturing of the joint capsule were performed, and an anti-adhesive agent based on hyaluronic acid (HA) (Guardix®, Hanmi Pharma., Seoul, Korea) was applied to the joint space to avoid adhesion within the space. Afterward, the overlying layers were meticulously closed for proper tissue healing as well as postoperative TMJ function. No surgical drain was inserted in the joint space. The skin was reapproximated and sutured with single interrupted No. 6-0 nylon sutures. Postoperative care was routinely performed according to our protocol. Briefly, intermaxillary fixation (IMF) was applied for 2 days after surgery, and early mouth opening exercises were initiated from 3 days after surgery. A soft diet was recommended for a month before the patient was allowed to gradually resume a normal diet. Wearing a prefabricated stabilization splint as soon as possible after the operation was advised, and occlusion in the patient was checked during each follow-up visit. Patients were instructed to continue the above conservative treatment practices throughout their life time.

### Evaluation criteria and statisticsok

All patients were operated on by the same surgeon (BK Lee), and all clinical evaluations were independently performed by three examiners. We applied the following evaluation criteria: (1) subjective TMJ pain using the visual analog scale (VAS) (0 = no pain, 10 = worst pain), (2) objective evaluation of: maximal mouth opening (MMO) measured as the distance between the upper and lower central incisal edges, and (3) TMJ noises during repeated opening of the jaw. In addition, the general success rate of our TMJ disc repositioning surgery technique using the MITEK mini anchor in this study was assessed according to the following criteria: (1) pain with VAS less than 1 at MMO or when chewing, (2) no or little TMJ noise with no discomfort in daily life, (3) MMO greater than 38 mm, and (4) no serious or permanent complications after surgery. The operation was regarded as a success when the patient fulfilled all three conditions at 6 months after the operation. Paired *t* tests were used for statistical analysis of the data in this study.

## Results

Sixty-five joints in 46 patients underwent unilateral or bilateral TMJ disc repositioning surgery with MITEK mini anchors. The following joint pathologies were diagnosed with MRI: ADDwoR, ADDwoR in association with joint effusion in 32 patients, ADDwR, ADDwR in association with joint effusion in 12 patients, and normal disc position in two patients who suffered from clinical symptoms like chronic pain and popping that were not resolved with conservative treatment.

**Fig. 10:**
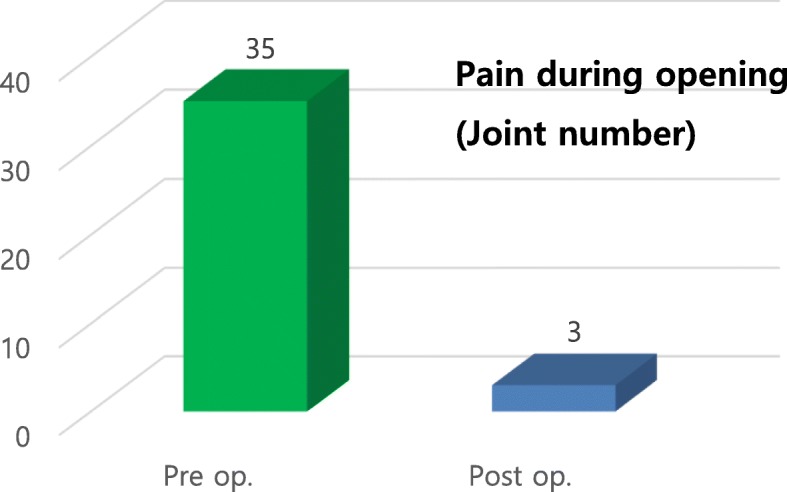
Evaluation of preoperative (Pre op.) and postoperative (Post op.) TMJ pain

**Fig. 11:**
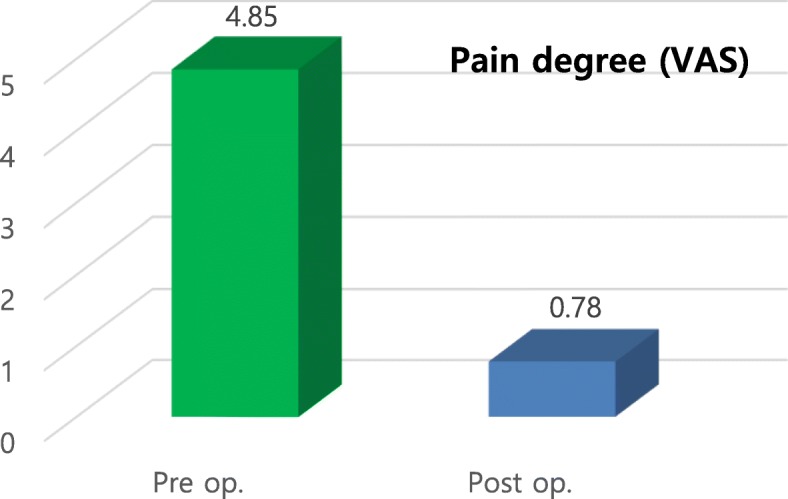
Evaluation of preoperative (Pre op.) and postoperative (Post op.) pain degree (VAS)

**Fig. 12:**
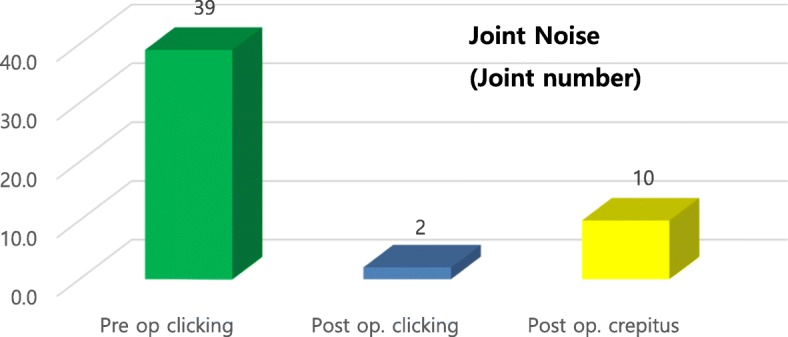
Evaluation of preoperative (Pre op.) and postoperative (Post op.) TMJ noise

**Fig. 13:**
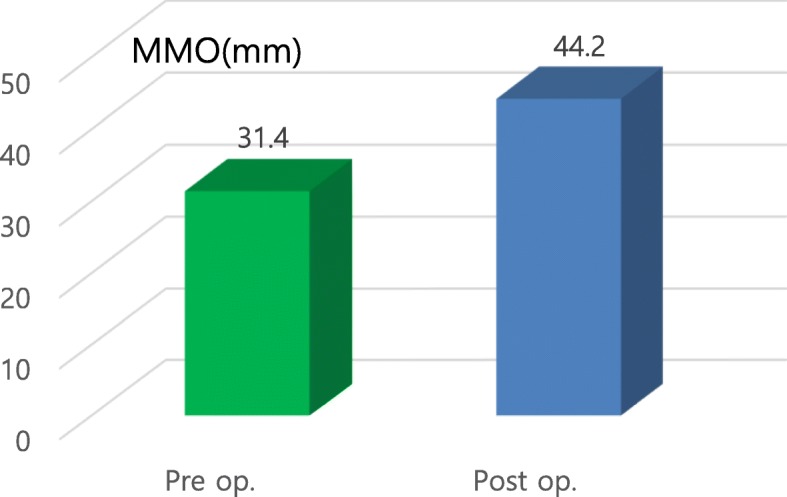
Evaluation of preoperative (Pre op.) and postoperative (Post op.) average MMO in trismus patients (*n* = 18)

Preoperative examination revealed painful symptoms in 50.7% (*n* = 35) of the evaluated joints (*n* = 69), with a mean VAS score of 4.85. Postoperative examination revealed that painful symptoms remained in 4.3% of joints (*n* = 3), with an average VAS score of 0.78, representing a 91.4% success rate in eliminating pain (Figs. 10 and 11). The presence of clicks in the TMJ was observed in 56.5% (*n* = 39) of joints postoperatively. Additionally, 17.4% (*n* = 12) of joints had residual noise on digital palpation in the TMJ, but only two joints made a clear clicking sound (Fig. 12), representing a 94.9% success rate with eliminating clicking. The clicking sounds in the other 10 TMJs were crepitus-like, with very little noise. In all 12 joints with residual noise, the intensity of the noise was significantly decreased compared to the preoperative condition. Interestingly, two out of the 10 crepitus-like residual noises were newly developed after the operation. The preoperative diagnosis for these two joints was anterior disc displacement without reduction with severe mouth opening limitation (MOL). Therefore, the newly developed noise resulted from increased movement of the condyle after the operation. Further, as observed in many cases, due to degeneration, TMJ discs that require surgical correction are likely to be already deformed and hardened compared to normal healthy discs. Therefore, the friction between the reposed disc and the articular fossa may be high, which may cause crepitus-like noise in the TMJ even without disc displacement from the condylar head. In patients with MOL less than 38 mm (*n* = 18), the mean MMO was 31.4 mm preoperatively and 44.2 mm at 6 months postoperatively (Fig. 13), with a mean increase of 13.8 mm. The MMO for patients who continued to visit our clinic for more than 6 months after surgery showed further increases over time until their last visit (2.2 mm in average). Six months after the operation, the number of patients with consistent follow-up decreased with time. Only 50% of patients (*n* = 23) continued their follow-up visits by 3 years after the operation. Cosmetically, the result was satisfactory because no visible scar was left at the operation site. Additionally, no significant complications such as facial nerve palsy or serious permanent occlusal disharmony were noted during the postoperative observation period. As a result, the general success rate of our TMJ disc repositioning surgery using MITEK mini anchors in this study was 91.0% (42/46) at 6 months after surgery.

## Discussion

Sufficient mouth opening, pain relief, functional stability, and long-term maintenance of the position of the disc are the main objectives of TMJ disc repositioning surgery [24]. Though disc repositioning surgery is often necessary to achieve these objectives, many surgeons are against the surgery because it can lead to the aggravation of TMJ symptoms, relapse of the disc, and potential side effects such as facial nerve damage and scars [25]. However, many efforts to overcome these hurdles have been made by some surgeons with technological advancements and better understanding of the pathophysiology of TMDs. Thus, more comprehensive approaches before and after disc plication surgery have been established. Basic principles of TMJ treatment should be applied before and after any surgical intervention, and all predisposing factors should be controlled with conservative treatment like self-administered physical therapy to relax masticatory muscles and other self-care behavior like avoiding bad habits including clenching and chewing habits. These conservative management practices should be observed throughout the patient’s life after surgery because various factors that contribute to disc displacement remain even after surgery. Factors that can predispose or cause TMJ disc displacement and dysfunction include trauma, parafunctional habits, gender, malocclusion, hormones, and systemic or local disease/pathology [1]. Discs can become displaced because of rupture, tearing, herniation, stretching, or degeneration of the ligaments that normally support the disc in position [1]. Therefore, management of predisposing factors is indispensable for the success of surgery and long-term stability [26].

The disc plication of TMJ using MITEK mini anchor is one of newly developed techniques but has been still improving to enhance its clinical outcome. Basically, proper diagnosis and meticulous surgery with minimal trauma are essential. In this sense, in this MITEK anchor technique, determination of the position and condition of the TMJ disc was critical to plan the surgical scheme prior to the operation. To date, the interpretation of MRI is essential for determining the disc position, amount of joint effusion, and bone abnormalities [27]. To ensure accurate diagnosis of TMJ disorders, the combination of MRI findings with clinical examination is critical [28]. The MITEK technique cannot be applied to all types of displaced TMJ discs. Because the anchor can only be inserted in a limited direction on the lateral or posterior surface of the condylar head, lateral or posterior displacement of the disc is not as effective as medial or anterior disc displacement.

As one of our important technical modifications, we cauterized the redundant retrodiscal tissue with electrocautery instead of cutting away the area as in the traditional MITEK procedure. This modification could avoid a huge retrodiscal dead space which may cause severe scar formation or adhesion of the joint space. Another unique feature of our modified method is that drainage is not inserted into the joints after surgery, but a commercial HA is injected into the superior joint cavity following capsular suture. This method can reduce the patient's discomfort when the drain is removed. It also has the advantage of not only alleviating inflammation in the joints due to the pharmacological action of the HA, but also preventing adhesion of the joints.

In all cases, we used the modified short endaural approach [29], which left a nearly invisible scar and provided enough space to place the MITEK anchors and reposition the disc. Esthetically, invisibility of the post-operative scar is essential for young female patients. As the proportion of female patients that undergo disc displacement of the TMJ is quite large, this approach has a certain benefit over the conventional surgical design for facial esthetics.

As our study showed, MITEK mini anchors were placed to facilitate repositioning of the joint disc over the condylar head, thus facilitating the physiologic movement and function of the joint structures. Our study results coincide with findings reported by other authors in terms of the mean age versus the clinical manifestations of dysfunctional TMJs. Our patients’ ages ranged between 15 and 69 years, with a mean age of 34.6 years, correlating with the study by Mehra and Wolford [1] where the mean age of patients was 32.6 years, with a range between 14 and 57 years. Our study population also coincides with that in the study by Sato et al [30], where the age range for patients who underwent surgery was between 16 and 45 years, with a mean age of 29.2 years, and to that of Anderson et al. [7], with a mean age of 28.1 years and a range between 14 and 48 years. Similarly, the distribution of gender in this study had a ratio of 1:1.7 (18 men vs 32 women), coinciding with the 1:1.8 male to female ratio reported in the literature, confirming the greater prevalence of TMJ disorders in female patients [1, 30, 31].

On the other hand, this study highlights important differences in symptoms before and after the procedure—postoperative absence of pain in 91.4% of patients, mean improvement in mouth opening by 13.8 mm in trismus patients, and absence of clicks in 92.9% of individuals evaluated, which correspond to other studies using MITEK mini anchors in the TMJ. Analyzing the presence of postoperative articular sounds, we found a correlation with the study by Mehra and Wolford [1], where the authors reported a 91% postoperative success rate, with improvements in pain, articular sounds, and mouth opening range. In addition, Fernandez Sanromán et al. [31] reported subjective TMJ pain improvement, with an increase in the mouth opening range on postoperative assessment. However, they reported persistent articular sounds in eight of the 12 patients included in their study, which is a higher incidence rate for postoperative articular sounds than in our study. Interestingly, habitual subluxation cases in this study showed excellent results during our observation period. Generally, habitual subluxation is caused by an imbalance of the disc and condylar head position due to laxation of the ligaments. In this sense, tightening the disc position and condylar head can restore balanced symbiotic movement in complex condylar movement of the mandible. Additionally, open TMJ surgery may lessen mandibular hypermobility, which is another benefit for managing subluxation.

**Fig. 14:**
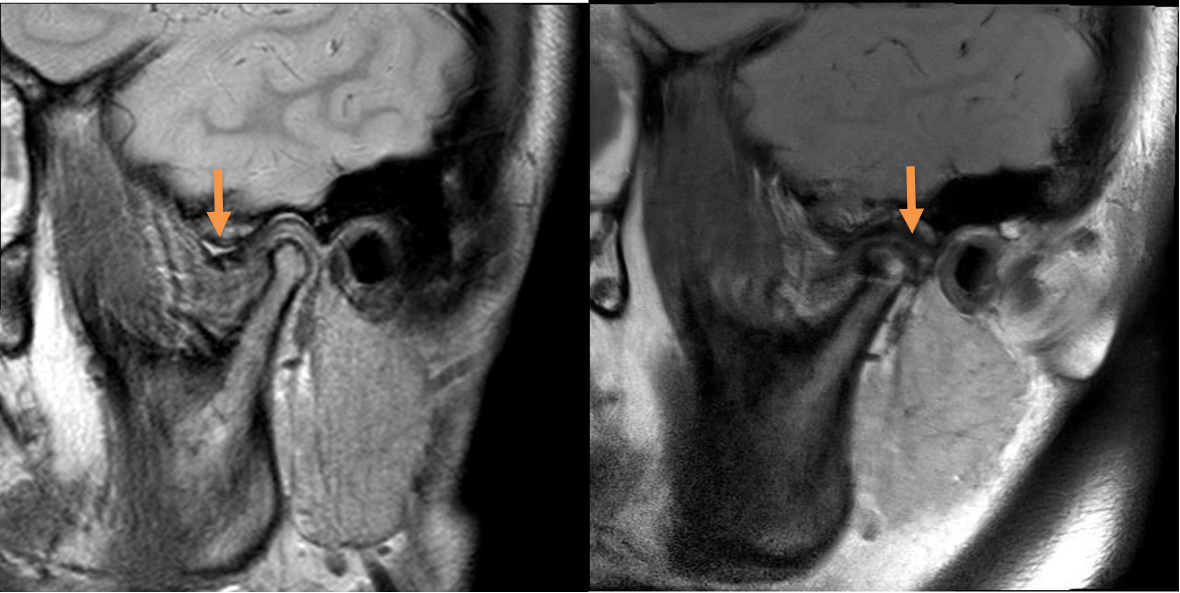
**a**, **b** Pre- and postoperative (1 year) MRI of a female patient who underwent left joint disc plication surgery. The disc position is marked with an arrow

In this study, unfortunately, post-operative MRI could not be performed for all patients because some of the patients refused to undergo MRI due to the high cost and the reduced discomfort in their TMJs. In addition, radiologic artifact of MITEK anchor in MRI could interfere the clear interpretation of the operated condyle in MRI (Fig. 14). Therefore, for most patients, post-operative disc positions were evaluated by digital palpation and the path of mouth opening. Nonetheless, this method was effective for diagnosing disc displacement [14].

Considering the noise, there were two cases of recurrence in this study. These patients did not follow our post-operative instructions properly and overused their TMJ. In particular, they were also under a lot of stress from their surrounding social environments. As is well known, chronic stress acts as a major contributor to tense masticatory muscles, leading to the inflammation of muscles and ligaments and causing or exacerbating jaw joint disorders. Therefore, for patients with excessive stress other than the jaw joint, it is advisable to suspend or defer disc plication surgery until their stress lessens to achieve good surgical results.

Temporary occlusal discrepancy after disc plication surgery is relatively common, but these changes normalize spontaneously over time without additional treatment like occlusal equilibration or orthodontic treatment.

## Conclusion

We conclude that our modification technique using MITEK mini anchors represent an alternative with great utility for procedures such as discopexy of the TMJ, showing excellent results in terms of improving function and patient quality of life. The improvements in postoperative pain, joint clicks, and mouth opening range are significant as long as the risk factors are reasonably managed and conservative treatment practices are continuously observed.

## Data Availability

All data generated or analyzed during this study are included in this published article.
